# Short-Wavelength and Near-Infrared Autofluorescence in Patients with Deficiencies of the Visual Cycle and Phototransduction

**DOI:** 10.1038/s41598-020-65763-x

**Published:** 2020-06-02

**Authors:** Jin Kyun Oh, Jose Ronaldo Lima de Carvalho, Joseph Ryu, Stephen H. Tsang, Janet R. Sparrow

**Affiliations:** 1 Department of Ophthalmology, Columbia University Medical Center, New York, NY USA; 2 State University of New York at Downstate Medical Center, Brooklyn, NY USA; 3 Department of Ophthalmology, Empresa Brasileira de Servicos Hospitalares (EBSERH) - Hospital das Clinicas de Pernambuco (HCPE), Federal University of Pernambuco (UFPE), Recife, Brazil; 4 Department of Ophthalmology, Federal University of São Paulo (UNIFESP), São Paulo, Brazil; 5 Department of Pathology & Cell Biology, Columbia University Medical Center, New York, NY USA

**Keywords:** Hereditary eye disease, Retinal diseases

## Abstract

Fundus autofluorescence is a valuable imaging tool in the diagnosis of inherited retinal dystrophies. With the advent of gene therapy and the numerous ongoing clinical trials for inherited retinal degenerations, quantifiable and reliable outcome measurements continually need to be identified. In this retrospective analysis, normalized and non-normalized short-wavelength (SW-AF) and near-infrared (NIR-AF) autofluorescence images of ten patients with mutations in visual cycle (VC) genes and nineteen patients with mutations in phototransduction (PT) genes were analyzed. Normalized SW-AF and NIR-AF images appeared darker in all patients with mutations in the VC as compared to patients with mutations in PT despite the use of significantly higher detector settings for image acquisition in the former group. These findings were corroborated by quantitative analysis of non-normalized SW-AF and NIR-AF images; signal intensities were significantly lower in all patients with mutations in VC genes as compared to those with mutations in PT genes. We conclude that qualitative and quantitative SW-AF and NIR-AF images can serve as biomarkers of deficiencies specific to the VC. Additionally, quantitative autofluorescence may have potential for use as an outcome measurement to detect VC activity in conjunction with future therapies for patients with mutations in the VC.

## Introduction

Fundus autofluorescence (AF) is a valuable imaging tool for both the diagnosis and monitoring of a variety of retinal diseases including age related macular degeneration, inflammatory retinal diseases, and in particular, inherited retinal dystrophies (IRDs)^1–5^. These conditions frequently present with observable phenotypes in AF images, such as the hyperautofluorescent ring seen in patients with retinitis pigmentosa (RP). These findings may be difficult to recognize in other forms of diagnostic imaging, such as fundus photography or spectral-domain optical coherence tomography (SD-OCT)^4^. The most common subtypes of fundus AF include short-wavelength fundus autofluorescence (SW-AF; 488 nm) and near-infrared fundus autofluorescence (NIR-AF; 787 nm).

The SW-AF signal (488 nm excitation) is emitted from the assemblage of bisretinoid fluorophores that are deposited in the retinal pigment epithelial (RPE) cells as lipofuscin. These deposits are formed as a result of non-enzymatic reactions of vitamin A aldehydes in photoreceptor cells^6^. In contrast, NIR-AF signal (787 nm excitation) is currently considered to be derived mostly from RPE melanin with smaller contributions from choroidal melanin^4,7^. To date, SW-AF has been used to monitor disease progression in a number of IRDs including recessive Stargardt disease (STGD1), RP, choroideremia, and Best vitelliform macular dystrophy^8–18^. NIR-AF is a more recently described imaging modality but has similarly been shown to be valuable in monitoring progression in STGD1, RP, and Best vitelliform macular dystrophy^10,13,15^.

The canonical visual cycle (VC) is a complex process involving the conversion of vitamin A (all -*trans*-retinol) into 11-*cis-*retinal in RPE cells; after transport to photoreceptor cells the latter chromophore binds to opsin. Phototransduction (PT) is initiated when light energy is used to isomerize 11-*cis*-retinal into all-*trans*-retinal. Bisretinoid fluorophores are produced from uncontrolled reactions of all-*trans*- or 11-*cis*-retinal, thus, it follows that vitamin A deficiency can cause a decrease in bisretinoid fluorophore production and result in a decrease in detected SW-AF signal^19^. This was confirmed in studies of both rats and humans exhibiting vitamin A deficiency; a marked decrease in the SW-AF signal has been demonstrated^20–22^. Similarly, disruptions of RPE65, the enzyme involved in the isomerization of all-*trans*-retinyl esters into 11-*cis*-retinol, have been shown to cause decreased SW-AF in humans and mice^23–25^.

In this study we find that SW-AF is decreased in normalized images acquired from patients with mutations in genes involved in the conversion of all-*trans*-retinol into 11-*cis*-retinal in RPE cells. These genes include *RPE65, LRAT, RLBP1*, and the 11-cis retinol dehydrogenases *RDH5* and *RDH11*. LRAT produces all-*trans-*retinyl ester which is then converted to 11-*cis*-retinol by RPE65. CRALBP, the protein product of the *RLBP1* gene, subsequently binds 11-*cis*-retinol and facilitates the oxidation of 11-*cis*-retinol into 11-*cis*-retinal by the 11-*cis*-retinol dehydrogenases, RDH5 and RDH11. 11-*cis*-retinal can then be transported to photoreceptor cells for photoisomerization into all-*trans*-retinal. RDH10 is an additional 11-*cis*-retinol dehydrogenase that causes delayed dark adaptation in the mouse model but has yet to be implicated in human retinal disease^26–28^.

In this study, the authors highlight the potential use of fundus AF not only as a first step in the elucidation of genes involved in specific inherited retinal dystrophies, but also in the potential use of quantitation of SW-AF and NIR-AF as an outcome measure in clinical trials^7–9^.

## Results

### Patients

Ten patients carrying mutations in VC genes (VC1-VC10) and nineteen patients with mutations in PT genes (PT1-PT19) were evaluated. Mean and median age at evaluation was 18.7 and 19 (range 9–27) years for patients in the VC cohort; 29.1 and 27 years (range 15–61) for patients in the PT cohort. Eight patients in the VC cohort and ten patients in the PT cohort were female. Demographic information is summarized in Supplemental Table 1 (seen online).

### Qualitative analysis of fundus images

**Figure 1:**
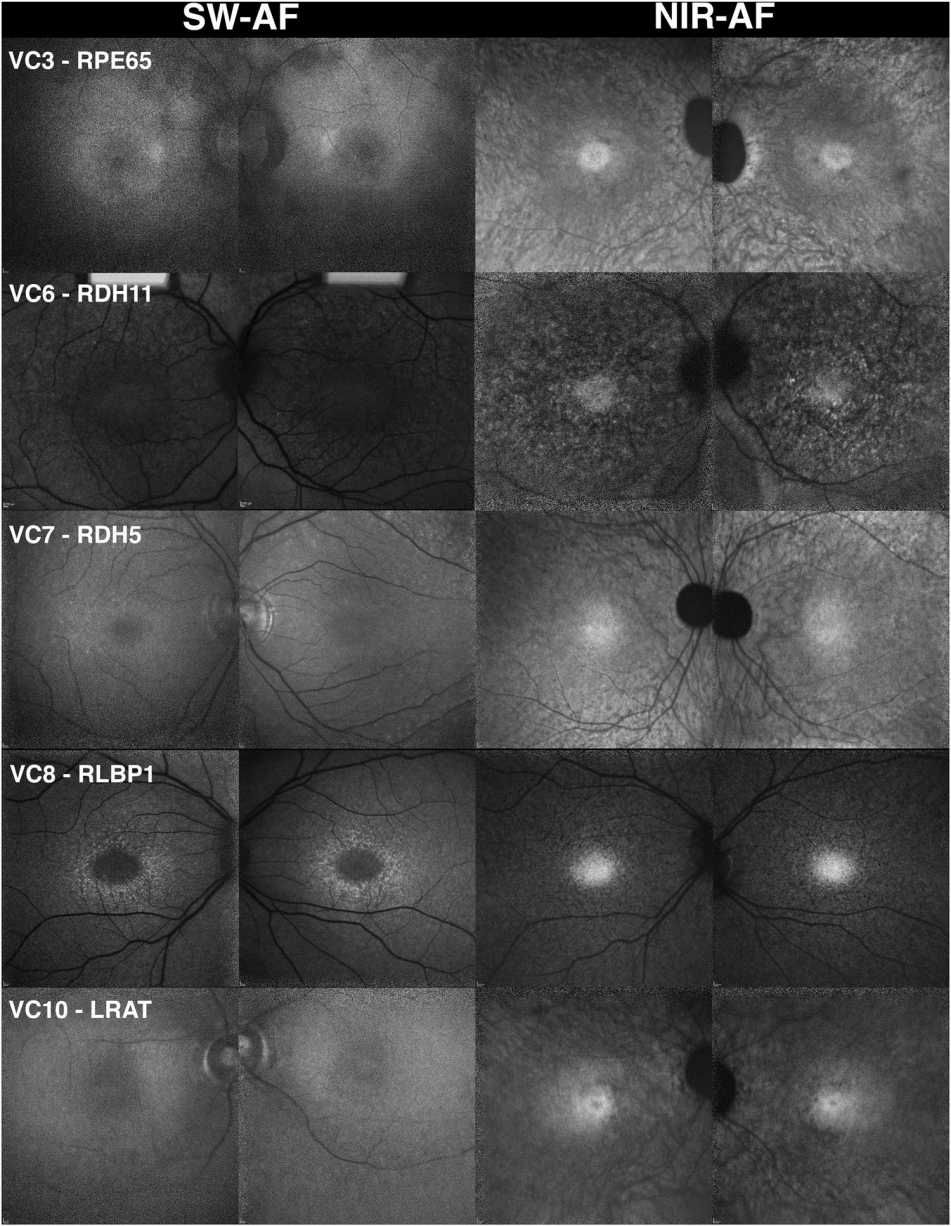
Fundus autofluorescence presenting in patients exhibiting mutations in visual cycle genes (VC). The short-wavelength autofluorescence (SW-AF) images of five patients carrying mutations in visual cycle genes, including *RPE65*, *LRAT*, *RLBP1*, *RDH5*, and *RDH11*, indicate that the feature common to this cohort is dark or absent autofluorescence illustrated by fluorescence intensity in the vasculature and optic nerve that is similar to the rest of the macula. Choroidal vessels are visible in the near-infrared autofluorescence (NIR-AF) images in the patients with mutations in *RPE65, LRAT*, and *RDH5*. This suggests a reduction in NIR-AF from RPE melanin. A hyperautofluorescent ring can be seen in the patient with mutations in *RLBP1* by short-wavelength autofluorescence. All other patients do not demonstrate hyperautofluorescent rings in either short-wavelength or near-infrared autofluorescence images.

**Figure 2:**
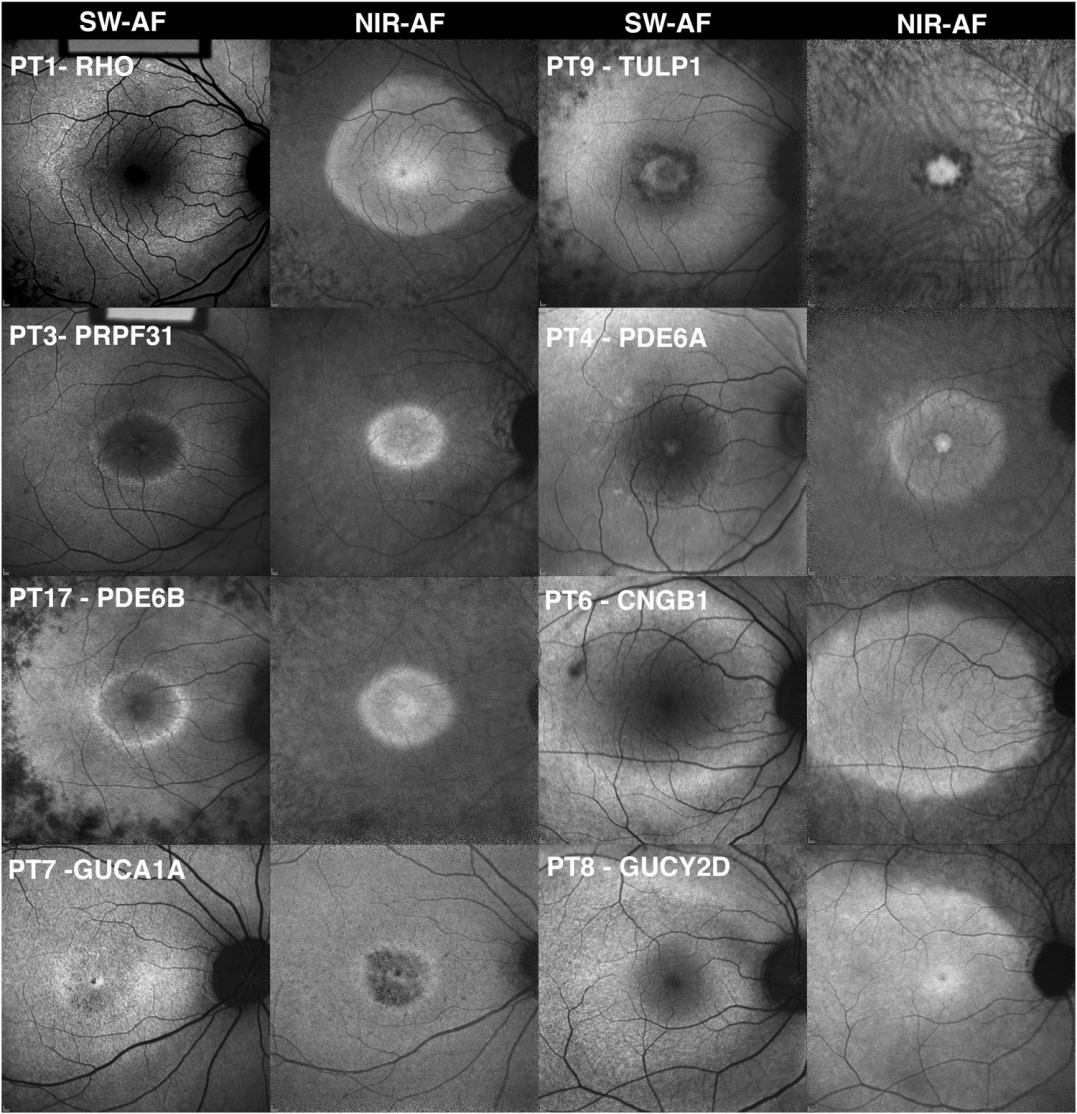
Fundus autofluorescence images obtained from patients with mutations in phototransduction genes (PT). The short-wavelength autofluorescence (SW-AF) and near-infrared autofluorescence (NIR-AF) images obtained from patients with mutations in phototransduction genes (*RHO, PRPF31, PDE6B, GUCA1A, TULP1, PDE6A, CNGB1*, and *GUCY2D)*, exhibit hyperautofluorescent rings in both SW-AF and NIR-AF images. The exception is the patient with a mutation in *GUCY2D*, who presents with a sectoral hyperautofluorescent arc in the superior retina. In all cases, autofluorescence was sufficient to enable imaging and the macula appeared brighter than the ophthalmic vessels and the optic nerve.

SW-AF (29 patients, 58 eyes), NIR-AF (23 patients, 46 eyes), and SD-OCT (29 patients, 58 eyes) images acquired from patients in the VC and PT cohorts were examined (Figs. 1 and 2). Qualitative evaluation of SW-AF images acquired from patients (20 eyes, 10 patients) carrying mutations in VC genes (Fig. 1) revealed vastly diminished autofluorescence signal when compared to those of patients with deficiencies in PT genes (38 eyes, 19 patients) (Fig. 2). In the former case, signal was weak despite the use of a higher sensitivity setting in the Heidelberg Spectralis HRA + OCT (p < 0.001) (see Supplemental Table S2). Specifically, images of patients in the VC cohort were acquired at mean and median sensitivities of 104 (range 100–107) while patients in the PT cohort were imaged at mean and median sensitivities of 85.8 and 88 respectively (range 74–100).The decrease in contrast of the signal associated with the optic disk and ophthalmic vasculature as compared to the rest of the macula is evidence of the hypoautofluorescence seen in the VC cohort relative to the PT cohort (Figs. 1 and 2).

NIR-AF images were acquired from 8 patients (VC1–3, VC5, VC7–10; 16 eyes) in the VC cohort. Five (10 eyes) were imaged using the HRA2 and a sensitivity setting of 96 (VC3, VC7–10; 10 eyes) while 3 patients (VC1–2, VC5; 6 eyes) were imaged using the HRA + OCT at a mean and median sensitivity of 97 (see Supplemental Table S2). Two patients, VC4 and VC6 did not undergo NIR-AF imaging. NIR-AF images were acquired from 15 patients (PT1, PT3–10, PT12–13, PT15–16, PT18–19; 30 eyes) in the PT cohort. Eleven were imaged using the HRA2 and a sensitivity of 96 (PT1, PT4, PT7–10, PT12–13, PT15–16, PT18; 22 eyes) while 4 patients (PT3, PT5–6, PT19; 8 eyes) were imaged using the HRA + OCT at a mean sensitivity of 99 (range 96–107). Four patients in the PT cohort, PT2, PT11, PT14 and PT17, did not undergo NIR-AF imaging. In contrast to the differences between the two groups in relation to autofluorescence intensities observed in SW-AF images, the NIR-AF images of the VC cohort did not demonstrate a qualitative difference in the strength of autofluorescence signal as compared to those of the PT cohort (Fig. 2).

A poorly defined, granular hyperautofluorescent ring was visible in SW-AF images of two patients (VC8 & VC9) in the VC cohort possessing mutations in *RLBP1* (VC8 illustrated in Fig. 1); rings were absent in the other eight patients. Hyperautofluorescent rings were not visible in NIR-AF images of any of the ten patients carrying mutations in VC genes (Fig. 1). In seventeen of the nineteen patients in the PT cohort, hyperautofluorescent rings were visible in both the SW-AF and NIR-AF modalities. Patients PT8 and PT9 did not have appreciable hyperautofluorescent rings. In NIR-AF images of 8 patients (VC1–3, VC5, VC7–10) exhibiting mutations in VC genes, choroidal vessels were visible in 6 patients. In each of the patients in the VC cohort, NIR-AF signal emitted at the fovea presented with sharp borders. In NIR-AF images of 15 patients (PT1, P3-PT10, PT12–13, PT15–16 PT18–19) exhibiting mutations in PT genes, choroidal vessels were visible in 9 patients. In each of the patients in the PT cohort, NIR-AF signal emitted at the fovea presented with sharp borders.

### Analysis of SW-AF intensities by quantitative fundus autofluorescence

**Figure 3:**
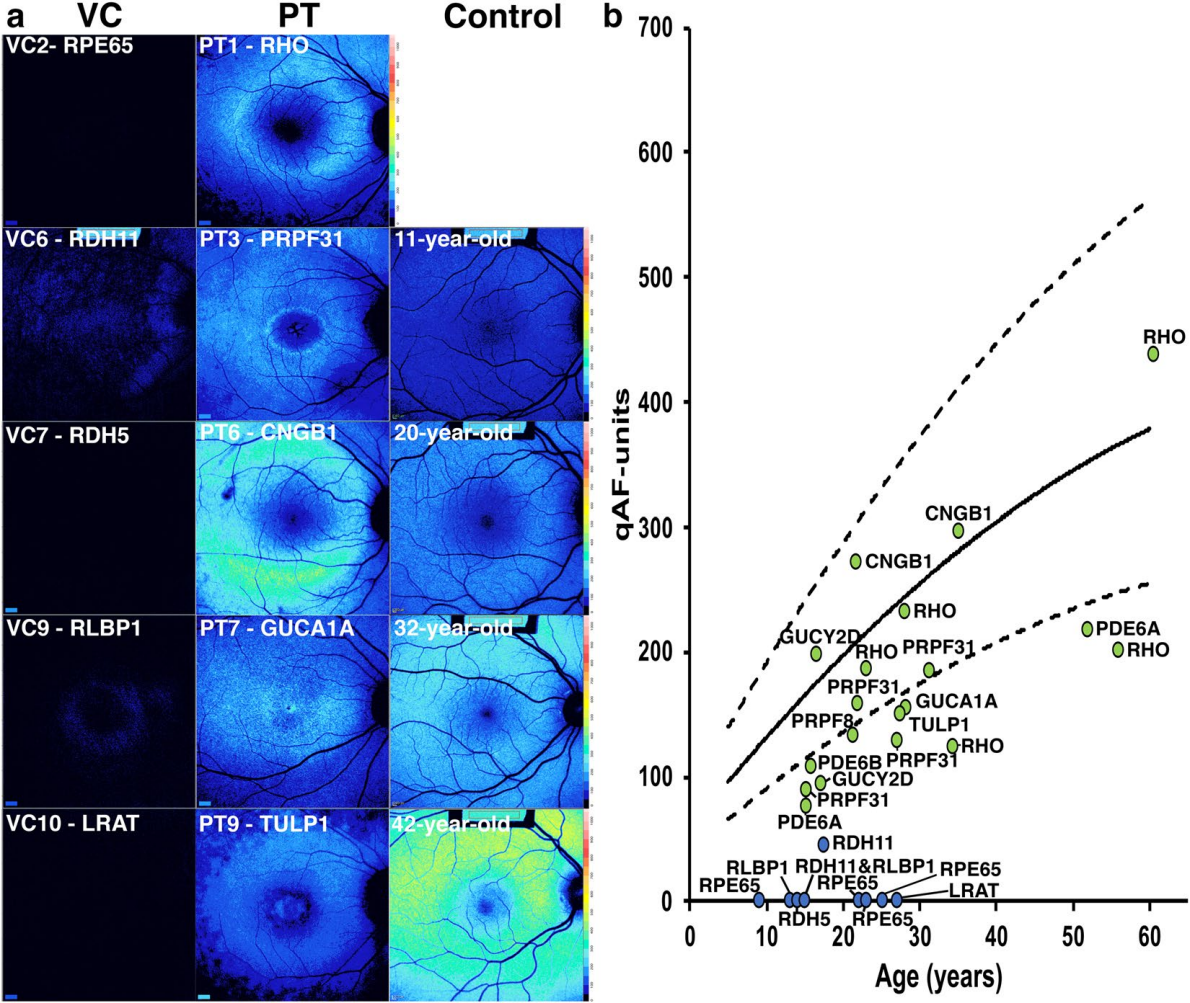
Quantitative fundus autofluorescence (qAF) in patients carrying mutations in visual cycle (VC) or phototransduction (PT) genes. (**a**) Representative qAF color maps acquired from patients carrying mutations in VC and PT genes together with age-matched healthy control subjects. Autofluorescence is very low or non-detectable in the presence of mutations in visual cycle genes. (**b**) qAF-unit values are plotted as a function of age in association with mutations in visual cycle genes (blue circles) and phototransduction genes (green circles). qAF-unit values acquired from patients with mutations in visual cycle genes are negligible and values for phototransduction genes are within the normal range or below normal regardless of age. Genes are indicated.

Quantitative autofluorescence (qAF) intensities associated with nine patients in the VC cohort (VC1–4, VC6–10) and nineteen patients in the PT cohort (PT1-PT19) was performed. Representative color-coded images of intensities scaled to qAF units (0–1200) for these two cohorts are illustrated in Fig. 3a alongside age-matched controls. These maps demonstrate the presence of sparse autofluorescence in patients with *RDH11* and *RLBP1* mutations (VC6 and VC9) and complete absence of autofluorescence in the other patients with mutations in VC genes. The qAF values are plotted as a function of age together with values obtained from normal age-matched controls (Fig. 3b).The qAF values associated with the patients in the VC cohort are negligible and fall far below the normal range of autofluorescence for healthy eyes, while qAF values acquired from the patients in the PT cohort fall either within the 95% confidence intervals or slightly below this normal range (Fig. 3b). This trend is seen even at older ages. Comparison of the mean qAF values of the VC and PT cohorts using Welch’s t-test revealed a statistically significant difference (p < 0.001).

**Figure 4:**
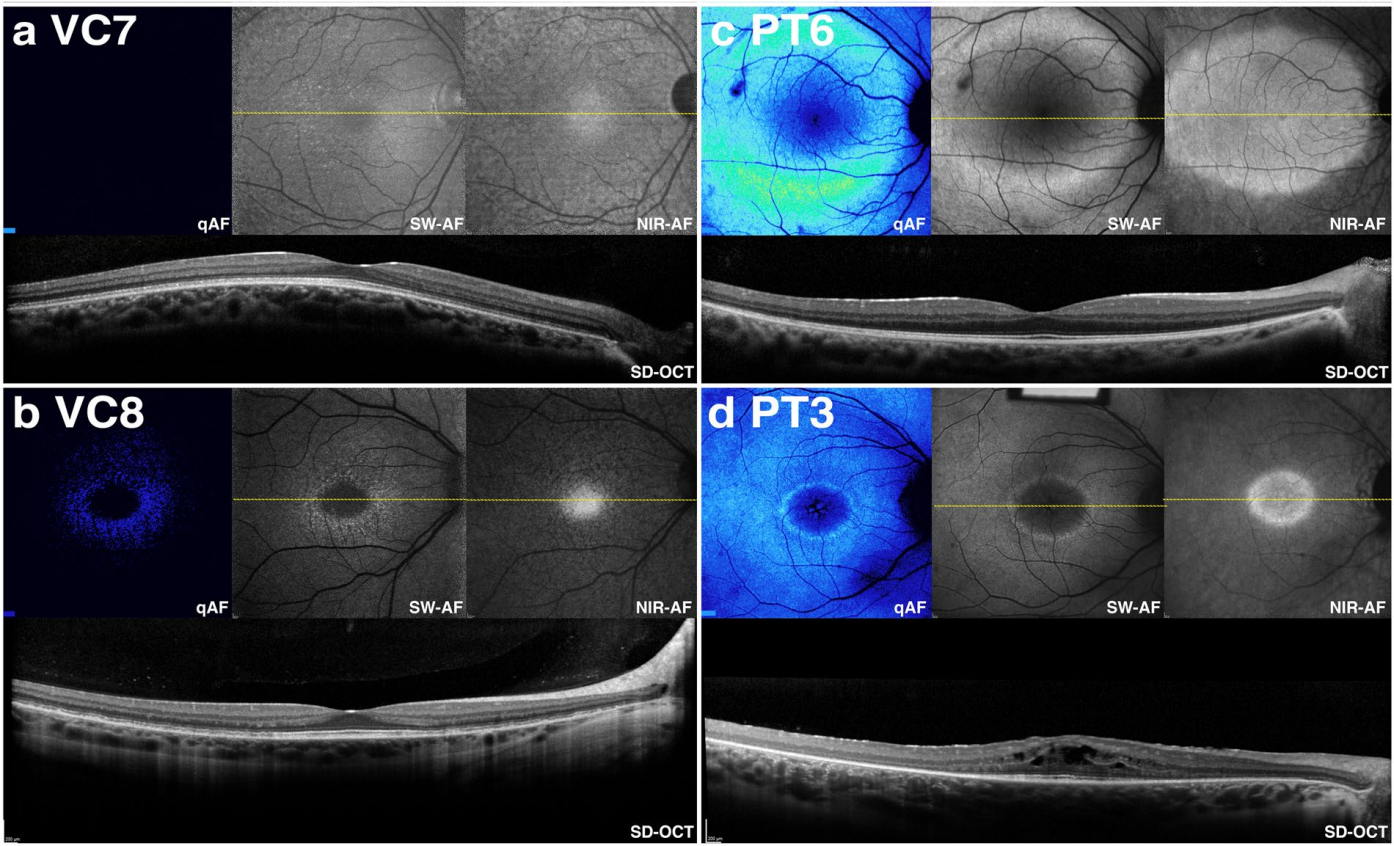
Comparison of color-coded quantitative fundus autofluorescence (qAF) with corresponding short-wavelength autofluorescence (SW-AF), near-infrared autofluorescence (NIR-AF), and spectral domain optical coherence tomographic (SD-OCT) images in patients carrying mutations in visual cycle (VC) (**a**, **b**; patients VC7 and VC8) or phototransduction (PT) (**c**, **d**; PT6 and PT3) genes. SD-OCT images indicate preserved outer retinal architecture as evidenced by lack of hypertransmission into the choroid. Note that qAF images have reduced AF signal in patients VC7 and VC8 as compared to patients PT6 and PT3.

We also examined the integrity of outer retina in the presence of reduced qAF. Examination of SD-OCT images obtained from patients in the VC cohort showed that in 3 of 9 patients, hypertransmission of SD-OCT signal into the choroid was not observed. For example as shown for patient VC7 (*RDH5*) in Fig. 4a, hypertransmission into the choroid was absent thus indicating that the RPE monolayer was intact. Conversely, patient VC8 (*RLBP1*) exhibited hypertransmission of SD-OCT signal in areas both temporal and nasal to the fovea (Fig. 4b). Irrespective of RPE integrity, both patients exhibited notable hypoautofluorescence when evaluated qualitatively. These levels were also lower than in patients in the PT cohort exhibiting either an intact RPE based on the absence of hypertransmission into the choroid (4/19 patients) (Fig. 4c, patient PT6) or an area of hypertransmission into the choroid (15/19 patients) (Fig. 4d, patient PT3).

### Quantitative Analysis of NIR-AF

**Figure 5:**
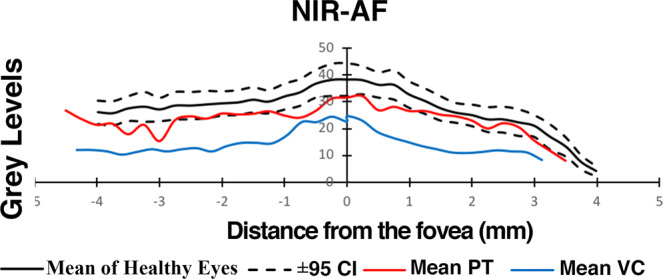
Semiquantitative analysis of near-infrared fundus autofluorescence (qNIR-AF) in patients carrying mutations in visual cycle (red line) (11 patients, 22 eyes) and phototransduction-specific (blue line) (5 patients, 10 eyes) genes. NIR-AF intensity profiles presented as mean grey levels are plotted as a function of temporal-to-nasal distance along a horizontal line (10 mm distance) through the fovea. Comparison is made to mean and 95% confidence intervals of healthy subjects (black lines) (19 controls, 38 eyes).

Semi-quantitative analysis of NIR-AF intensities obtained from five patients in the VC cohort (VC3, VC7–10) and eleven patients in the PT cohort (PT1, PT4, PT7–10, PT12–13, PT15–16, PT18) was performed (Fig. 5). Unlike the qualitative comparison of NIR-AF images which did not suggest a noticeable difference in autofluorescence signal strength between the VC and PT groups, quantitative analysis demonstrated that there was a significant difference in NIR-AF signal strength, with patients in the VC cohort demonstrating lower signal as compared to patients in the PT cohort. The NIR-AF values of patients in the PT cohort were similar to the lower 95% confidence interval of the normal range while those in the VC cohort were significantly below normal along the entire 10 mm horizontal distance that was analyzed. Individual patient data can be found in Supplemental Fig. S1 (seen online).

## Discussion

Fundus autofluorescence is a clinical imaging modality used for the evaluation of a wide spectrum of retinal disorders. In this study, we compared SW-AF and NIR-AF findings in two cohorts of patients with gene mutations in either the VC or the PT process.

Qualitative evaluation of SW-AF images indicated that dark autofluorescence is a feature common to patients with mutations in the VC genes. These VC genes are all specific to the RPE. In contrast, all of the patients with mutations in PT had appreciable autofluorescence with defining features characteristic of IRD, including the hyperautofluorescent ring typically seen in RP. Differences in autofluorescence associated with operator-dependent sensitivity settings were analyzed and showed that higher detector sensitivities were used to acquire images for the VC cohort as compared to those for the PT cohort (p < 0.001). This indicates that the difference in qualitative autofluorescence was not due to image acquisition settings. Analysis by qAF further corroborated the clinical findings in patients of the VC cohort; there was an absence or profound reduction of SW-AF signal as indicated by color maps and by plotting qAF values together with values for healthy eyes. A comparison of the images from patients with either intact or atrophic RPE from both cohorts suggests that these differences are not due to differences in RPE integrity. These findings are consistent with the hypothesis that disruptions of the VC lead to decreased production or absence of 11-*cis* chromophore and a resultant decrease in bisretinoid formation.

In contrast, patients in the PT cohort were found to have qAF intensities within the range of the mean and 95% confidence intervals for healthy eyes or below the 95% confidence interval. The gene mutations identified in these patients cause disruptions in photoreceptor function but do not interfere with production of 11-*cis* chromophore, and consequently do not have a direct effect on fundus autofluorescence. The cause of decreased autofluorescence signal in some patients in this cohort relative to the control group may be explained by photoreceptor death and, thus, decreased bisretinoid production. However, the issue with respect to the PT genes may be more complex. For instance, SW-AF originates from the unwanted bisretinoid compounds that form due to reactivity of retinaldehyde. What is not known is whether rhodopsin mutations that cluster around the 11-*cis*-retinal binding pocket affect the rate of bisretinoid formation from released retinaldehyde.

While a qualitative difference in the NIR-AF signal between the two cohorts was not appreciated, semi-quantitative analysis of NIR-AF using non-normalized images indicated that a significant difference existed between the two groups, with NIR-AF signal in the PT cohort being at the lower limit of the 95% confidence interval for healthy eyes while patients presenting with mutations in VC genes exhibited qNIR-AF intensities below the 95% confidence interval. The difference in NIR-AF signal is less easily explained than the difference in SW-AF signal, given that most of the NIR-AF signal is proposed to come from melanin pigment present in the RPE cells^6,10^. All of the VC genes discussed in this study are expressed by the RPE. Therefore, one explanation for the difference in NIR-AF seen between these two cohorts is the possibility of RPE cell changes in patients with mutations in the VC genes. For instance the diminished optical density of melanin may be attributable to RPE thinning as remaining cells spread and reorganize in response to RPE loss^10,11,29^. Further studies are warranted to better understand the reason for decreased NIR-AF signal concurrent with interruptions of vitamin A metabolism.

Consequently, the difference in autofluorescence signal between the two cohorts suggests that when clinicians encounter the combination of dark SW-AF images and recordable NIR-AF intensities in patients presenting with clinical features indicative of IRD, suspicion for VC gene mutations should be considered. Conversely, when both SW-AF and NIR-AF signal can be recorded at typical sensitivities, the likelihood that the mutated gene is involved in the vitamin A cycle is low. With the advent of treatment for IRDs caused by mutations in the visual cycle gene *RPE65*, clinicians should be vigilant with respect to this finding^30^.

In addition to the differences in SW- and NIR-AF signal seen between the two cohorts, a difference in the incidence of hyperautofluorescent rings was also observed. Only two patients with *RLBP1* mutations demonstrated the presence of a ring on SW-AF, but these rings were not apparent in NIR-AF images. In contrast, the other patients in the VC cohort did not have appreciable rings in either SW-AF nor NIR-AF images. The presence of the hyperautofluorescent ring in both patients with mutations in *RLBP1* and the absence of the ring in the other patients with mutations in the VC may be explained by the function of *RLBP1*. While the other visual cycle genes are directly responsible for 11-*cis* chromophore production, *RLBP1* augments RPE65 function, facilitates oxidation of 11-cis-retinol and maintains the 11-cis configuration but 11-cis-retinal chromophore can still be produced, albeit at reduced efficiency^31^. Consequently, mutations in *RLBP1* may decrease or delay 11-*cis-*chromophore production, but do not lead to complete loss^32,33^. This may explain the presence of minimal qAF signal in the qAF color map (Fig. 3a) together with the detection of a hyperautofluorescent ring in SW-AF images despite the ring being absent in other patients having mutations in VC genes.

In comparison, seventeen of the nineteen patients in the PT cohort exhibited a hyperautofluorescent ring in both SW-AF and NIR-AF images. The difference in the prevalence of this ring between the two groups is notable, as changes in the area, perimeter, horizontal diameter, and vertical diameter of the ring have been used in the past and continue to be proposed as outcome measurements in clinical trials for IRD^34,35^. These findings suggest that whereas AF rings in SW-AF and NIR-AF images may be suitable for use in evaluating therapies related to PT gene mutations, they may not serve to measure therapeutic outcomes when the mutations are in VC genes. On the other hand, qAF could potentially be used to detect visual cycle activity following gene therapy for patients with mutations in VC genes. Recovery of autofluorescence when measured by qAF was recently described in patients with vitamin A deficiency after treatment, suggesting that a similar finding may be noted after treatment of IRDs^21^. Altogether, this study extends the clinical utility of SW-AF and NIR-AF in the diagnosis, monitoring, and future treatment for gene mutations in proteins of the VC as opposed to gene mutations associated with PT.

## Methods

### Subjects

Retrospective chart review was performed to identify patients with homozygous or compound heterozygous mutations in *RPE65, LRAT, RLBP1, RDH5*, and *RDH11*. Eleven patients were identified who had confirmed mutations in these genes. From these 11 patients, one was excluded due to retina-wide atrophy on AF imaging, leaving 10 patients who fit inclusion criteria. Retrospective chart review was also performed to identify patients with confirmed mutations in the following PT specific genes: *RHO, CNGB1, PDE6A, PDE6B, GUCA1A, GUCY2D, PRPF31*, and *TULP1*. Seventy-five patients were identified who had confirmed mutations in these genes. From these 75 patients, those with panretinal atrophy on autofluorescence imaging or absence of quantitative autofluorescence images were excluded. In the case of multiple patients being identified within the same family, the youngest affected individual fitting the above criteria was selected, leaving a total of 19 patients who fit inclusion criteria. All 29 patients were evaluated at the Edward S. Harkness Eye Institute at Columbia University Medical Center (New York, NY). The study was conducted under Columbia University Institutional Review Board approval (protocol AAAR8743) and all procedures were in accordance with the tenets of the Declaration of Helsinki. Informed consent was waived as per protocol AAAR8743 due to the retrospective nature of the study design and the minimal risk conferred to patients.

### Ophthalmic examination & fundus imaging

Examination of patients was performed to determine best corrected visual acuity followed by dilation with topical tropicamide (1%) and phenylephrine hydrochloride (2.5%). Fundus examination was followed by imaging, including SD-OCT, SW-AF (488 nm excitation, barrier filter transmitted light from 500 to 680 nm, 30° × 30°), and NIR-AF (787 nm excitation, 830 nm emission, 30° × 30°). SD-OCT and SW-AF were acquired using a Spectralis HRA + OCT (Heidelberg Engineering, Heidelberg, Germany). NIR-AF was acquired using either the Spectralis HRA + OCT or Spectralis HRA2 (Heidelberg Engineering, Heidelberg, Germany). The images were acquired using sensitivity settings that optimized image quality and were saved in normalized mode. Statistical comparison of the sensitivities used for SW-AF and NIR-AF images was performed using Welch’s t-test and descriptive statistics. Analysis was performed using R statistical software version 3.6.1 (Vienna, Austria).

#### Acquisition and quantitation of SW-AF images

qAF was performed, as previously described using images acquired using a Spectralis HRA + OCT that is equipped with an internal fluorescent reference used to correct for variations in detector sensitivity and laser power^36,37^. First, a near-infrared reflectance image (NIR-R) was obtained. Afterwards, the fundus was exposed to the blue (488 nm) excitation mode for 20 seconds to facilitate a uniform signal. Subsequently, the qAF mode was selected and the camera was centered and focused at the fovea. Images were acquired in high-speed setting (8.9 frames/s) in video format at a minimum of 12 total frames per acquisition. Each acquisition was evaluated for quality, requiring consistency in at least 7 of the 12 total frame images. Images with frame misalignment due to movement or decreased AF signal due to eyelid interference were excluded. An average non-normalized image was produced from each video, and two images from two sessions were analyzed for a total of four averaged images per eye.

These images were exported to IGOR (WaveMetrics, Lake Oswego, OR, USA), and mean gray levels (GL) were calculated in three concentric rings composed of eight circular segments at eccentricities of 7° to 9° from the fovea. The effects of the vasculature and atrophic areas in the sampling area on qAF levels were excluded by histogram analysis. qAF values were calculated after GLs were calibrated to GLs in the reference and after accounting for zero-GL of the laser, refraction, image magnification, and age-adjusted lens transmission. A cohort of 277 patients with healthy eyes (age range 5 to 60) was used as control.

#### Acquisition and analysis of NIR-AF images

Non-normalized NIR-AF fundus images for semi-quantitative analysis were obtained using the Spectralis HRA2 in a 30° × 30° field with a fixed sensitivity of 96. The non-normalized images were exported and analyzed using ImageJ software (ImageJ, U.S. National Institutes of Health, Bethesda, Maryland, USA). Using NIR-AF images available from 11 patients in the PT cohort and 5 patients in the VC cohort, GL values were plotted for each patient as a function of distance from the fovea at 0.25 mm intervals. A control group of nineteen subjects with healthy eyes (mean age 35.96 years) was used as reference. Mean GLs (+/−95% confidence intervals) calculated at each position relative to the fovea were also plotted for the PT and VC cohorts and controls.

## Supplementary information

Supplemental Figure S1.

Supplemental information.

## Data Availability

All data generated or analysed during this study are included in this published article (and its Supplementary Information files).
