# Supplementary material for: Short-Wavelength and Near-Infrared Autofluorescence in Patients with Deficiencies of the Visual Cycle and Phototransduction
Source: Sci Rep. 2020 Jun 2;10:8998. doi: 10.1038/s41598-020-65763-x (PMC7265524; doi:10.1038/s41598-020-65763-x)
Supplement: Supplementary file 1 — Supplemental Figure S1. [file 41598_2020_65763_MOESM1_ESM.pdf]

Short-Wavelength and Near-Infrared Autofluorescence in Patients with Deficiencies of the Visual Cycle and Phototransduction

Jin Kyun Oh[1,2], Jose Ronaldo Lima de Carvalho Jr[1,3,4], Joseph Ryu[1], Stephen H. Tsang[1,5], Janet R. Sparrow[1,5§].

[1]Department of Ophthalmology, Columbia University Medical Center, New York, NY, USA
[2] State University of New York at Downstate Medical Center, Brooklyn, NY, USA
[3] Department of Ophthalmology, Empresa Brasileira de Servicos Hospitalares (EBSERH) - Hospital das Clinicas de Pernambuco (HCPE), Federal University of Pernambuco (UFPE), Recife, Brazil
[4] Department of Ophthalmology, Federal University of São Paulo (UNIFESP), São Paulo, Brazil.
[5] Department of Pathology & Cell Biology, Columbia University Medical Center, New York, NY, USA
[§] Corresponding author

Corresponding Author and Address for reprints:
Janet R. Sparrow PhD
Harkness Eye Institute, Columbia University Medical Center
630 West 168th Street, New York, NY 10032
Phone: (212) 305-9944 / Email: jrs88@cumc.columbia.edu

Supplemental Table 1. Demographics information of patients with visual cycle-gene and phototransduction-gene associated retinal dystrophy

Patient ID/ Gender	Age at diagnosis (years)	Age at imaging (years)	Gene and variants	BCVA	Ethnicity	Ring on SW-AF	Ring on NIR-AF	Retinal Findings
VC1/F	5	22	RPE65 (c.1205G>A:p.Tr402Ter) (c.1022T>C:p.Leu341Ser)	20/400, 20/400	Hispanic	No	No	Minimal intraretinal pigment migration
VC2/F	6	25	RPE65 (c.1205G>A:p.Tr402Ter) (c.1022T>C:p.Leu341Ser)	20/400, 20/250	Hispanic	No	No	Minimal intraretinal pigment migration
VC3/M	4	23	RPE65 (c.65T>C:p.Leu22Pro) (c.400_402delGTT:p.Val134del)	20/70, 20/70	Brazilian	No	No	Minimal intraretinal pigment migration
VC4/F	0.5	9	RPE65 (p.Lys295Ter) (c.202C>T:p.His68Tyr)	20/400, 20/150	Caucasian	No	No	RPE Mottling
VC5/M	13	13	RDH11 (c.199C>T:p.Arg67Ter) (c.322C>T:p.Arg108Ter)	20/40, 20/40	Caucasian	No	No	Mottled macula
VC6/F	15	15	RDH11 (c.199C>T:p.Arg67*) (c.322C>T:p.Arg108*)	20/40, 20/40	Caucasian	No	X	Mottled Macula
VC7/F	15	14	RDH5 (c.160C>T:p.Arg54Ter) (c.572G>A:p.Arg191Gln)	20/30, 20/30	Hispanic	No	No	Fine subretinal dots

**VC8/F**	**12.5**	**15**	***RLBP1*** ***(c.286_297del:p.Phe96_Phe99)*** ***(c.25C>T:p.Arg9Cys)***	**20/20, 20/20**	**Caucasian**	**Yes**	**No**	**RPE mottling**
**VC9/F**	**11.5**	**13**	***RLBP1*** ***(c.286_297del:p.Phe96_Phe99)*** ***(c.25C>T:p.Arg9Cys)***	**20/20, 20/30**	**Caucasian**	**Yes**	**No**	**RPE mottling**
**VC10/F**	**4**	**27**	***LRAT*** ***(c.459dupC:p.Tyr154LeufsX30*** ***(Whole gene deletion of exons 1-3)***	**20/CF, 20/CF**	**Caucasian**	**No**	**No**	**RPE mottling**
**PT1/F**	**14**	**16**	***RHO (c.937-27_-19delCCCTGATCTC)***	**20/20, 20/20**	**Caucasian**	**Yes**	**Yes**	**Intraretinal pigment migration**
**PT2/M**	**21**	**26**	***PRPF8*** ***(c.4022+15A>G)***	**20/20, 20/20**	**Caucasian**	**Yes**	**Yes**	**Intraretinal pigment migration**
**PT3/F**	**17**	**17**	***PRPF31*** ***(c.383T>A:p.Leu128Ter)***	**20/40, 20/25**	**Caucasian**	**Yes**	**Yes**	**No Intraretinal pigment migration**
**PT4/M**	**14**	**14**	***PDE6A*** ***(c.1705C>A:p.Gln569Lys)*** ***(c.2263C>T:p.Gln755Ter)***	**20/30, 20/30**	**Caucasian**	**Yes**	**Yes**	**Intraretinal pigment migration**
**PT5/F**	**23**	**28**	***RHO*** ***c.50C>T (p.Thr17Met)***	**20/20 20/20**	**Caucasian**	**Yes**	**Yes**	**Intraretinal pigment migration**
**PT6/M**	**16**	**17**	***CNGB1*** ***(c.3150delG: pPhe1051Leufs*12)*** ***(c.3150delG: pPhe1051Leufs*12)***	**20/25, 20/20**	**Hispanic**	**Yes**	**Yes**	**No Intraretinal pigment migration**
**PT7/F**	**19**	**28**	***GUC1A1*** ***(c.296A>G:p.Tyr99Cys)***	**20/100, 20/100**	**Asian**	**Yes**	**Yes**	**No Intraretinal**

								pigment migration
PT8/F	15	23	*GUCY2D (c.3220C>T:p.Pro1074Ser)*	20/20, 20/20	African American	No	No	Intraretinal pigment migration
PT9/M	12	26	*TULP1 (c.349G>A:p.Glu117Lys) (c.349G>A:p.Glu117Lys)*	20/80, 20/50	Asian	No	No	Intraretinal pigment migration
PT10/M	14	16	*GUCY2D (c.2512C>T:p.Arg838Cys)*	20/20, 20/30	Caucasian	Yes	Yes	No Intraretinal Pigment Migration
PT11/F	15	15	*PRPF31 (c.856-2A>G)*	20/20, 20/40	Caucasian	Yes	Yes	No Intraretinal Pigment Migration
PT12/M	26	27	*PRPF31 (c.73G>T:p.Glu25Ter)*	20/20, 20/25	Caucasian	Yes	Yes	Intraretinal pigment migration
PT13/M	25	31	*PRPF31 (c.322+4_322+7delAGTG)*	20/80, 20/25	Hispanic	Yes	Yes	Intraretinal pigment migration
PT14/F	56	56	*RHO (c.68C>A:p.Pro23His)*	20/40, 20/40	Caucasian	Yes	Yes	Intraretinal Pigment Migration
PT15/F	34	34	*RHO (c.1040C>T:p.Pro347Leu)*	N/A	Caucasian	Yes	Yes	Intraretinal Pigment Migration
PT16/M	61	61	*RHO (c.68C>A:p.Pro23His)*	N/A	Caucasian	Yes	Yes	Intraretinal Pigment Migration

**PT17/M**	**13**	**16**	***PDE6B* (*c.1923_1969ins6del47:p. Tyr641TfsX31*) (*c.1923_1969ins6del47:p. Tyr641TfsX31*))**	**20/25, 20/25**	**Caucasian**	**Yes**	**Yes**	**Intraretinal Pigment Migration**
**PT18/F**	**46**	**51**	***PDE6A* (*c.1250A>G:p.Glu417Gly*) (*c.1250A>G:p.Glu417Gly*)**	**20/60, 20/70**	**Hispanic**	**Yes**	**Yes**	**Intraretinal Pigment Migration**
**PT19/F**	**27**	**36**	***CNGB1* (*c.3150delG:p.Phe1051Leufs*12*) (*c.1941C>A:p.647Arg*)**	**20/20, 20/25**	**Brazilian**	**Yes**	**Yes**	**Intraretinal Pigment Migration**

VC – patients carrying mutations in visual cycle genes, PT – patients carrying mutations in phototransduction genes, BCVA – best corrected visual acuity, SW-AF – short-wavelength autofluorescence, NIR-AF – near-infrared autofluorescence

Supplemental Table 2. Sensitivities for Short-Wavelength and Near-Infrared Autofluorescence Imaging Acquisition

Patient ID	SW-AF	NIR-AF
VC1	100	96 (HRA+OCT)
VC2	104	96 (HRA+OCT)
VC3	106	96 (HRA2)
VC4	107	x
VC5	102	98 (HRA+OCT)
VC6	100	x
VC7	103	96 (HRA2)
VC8	107	96 (HRA2)
VC9	107	96 (HRA2)
VC10	102	96 (HRA2)
PT1	89	96 (HRA2)
PT2	74	x
PT3	78	96 (HRA+OCT)
PT4	87	96 (HRA2)
PT5	89	97 (HRA+OCT)
PT6	69	96 (HRA+OCT)
PT7	90	96 (HRA2)
PT8	91	96 (HRA2)
PT9	78	96 (HRA2)
PT10	91	96 (HRA2)
PT11	85	x
PT12	94	96 (HRA2)
PT13	96	96 (HRA2)
PT14	92	x
PT15	94	96 (HRA2)
PT16	88	96 (HRA2)
PT17	82	x
PT18	88	96 (HRA2)
PT19	76	107 (HRA+OCT)

VC – patients carrying mutations in visual cycle genes, PT – patients carrying mutations in phototransduction genes, SW-AF – short-wavelength autofluorescence, NIR-AF – near-infrared autofluorescence

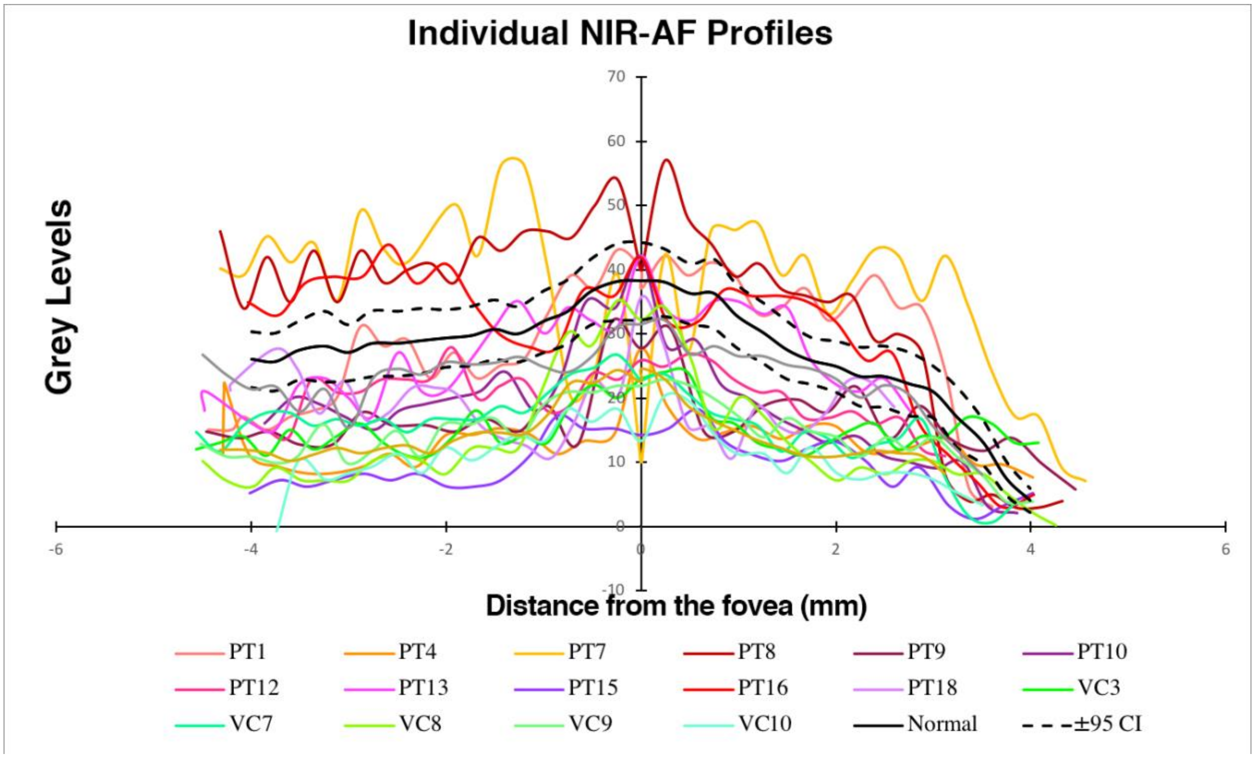

Supplemental Figure 1. Semi-quantitative analysis of near-infrared fundus autofluorescence (qNIR-AF). Profiles are plotted for individual patients carrying mutations in phototransduction (PT) and visual cycle (VC) genes. Graphs of the semiquantitative analysis of each individual patient suggesting the common feature of normal near-infrared autofluorescence in all patients with mutations in phototransduction genes and hypoautofluorescence in patients with mutations in visual cycle genes.
