# Supplementary figures and images for: Short-Wavelength and Near-Infrared Autofluorescence in Patients with Deficiencies of the Visual Cycle and Phototransduction
Source: Sci Rep. 2020 Jun 2;10:8998. doi: 10.1038/s41598-020-65763-x (PMC7265524; doi:10.1038/s41598-020-65763-x)

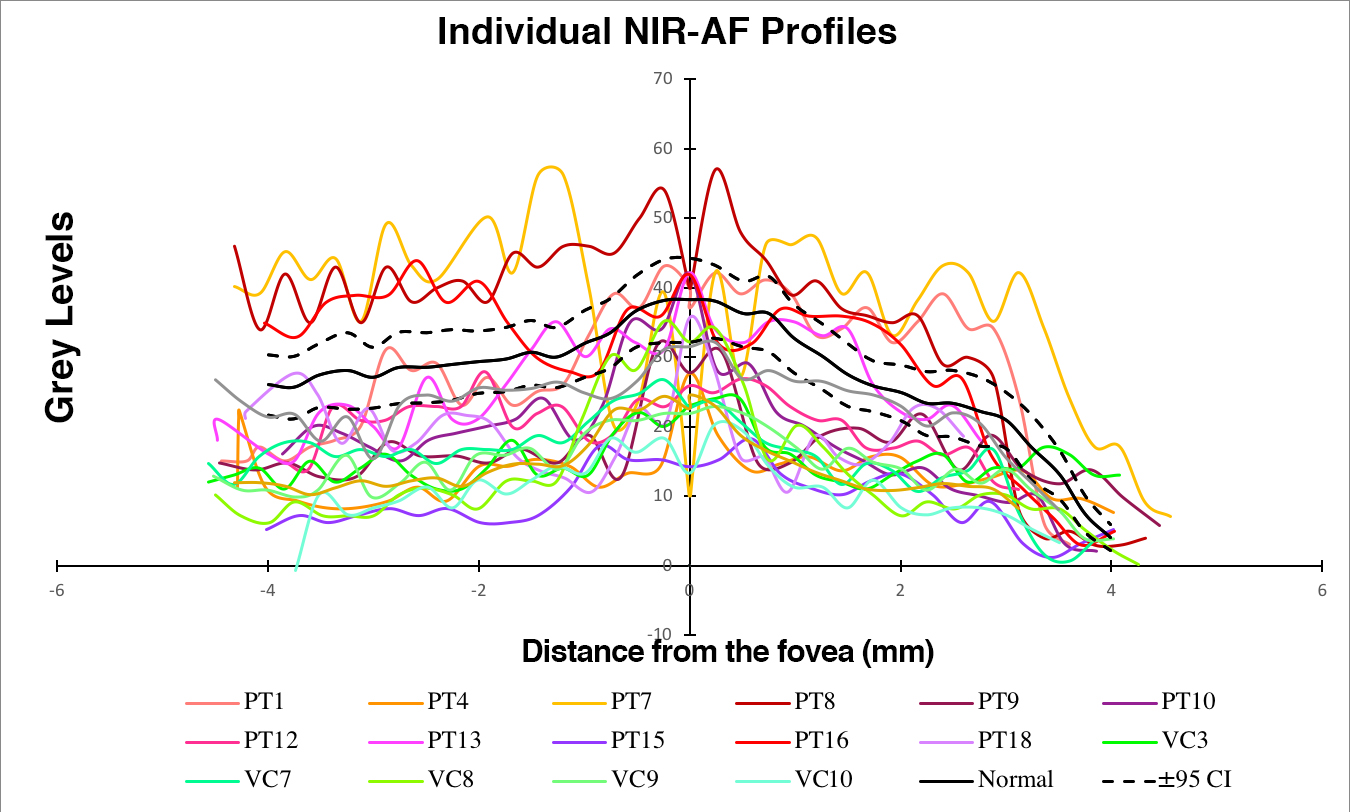

Supplement: Supplementary file 2 — Supplemental information. [file 41598_2020_65763_MOESM2_ESM.jpg]
